# A novel approach to quantify metrics of upwelling intensity, frequency, and duration

**DOI:** 10.1371/journal.pone.0254026

**Published:** 2021-07-08

**Authors:** Amieroh Abrahams, Robert W. Schlegel, Albertus J. Smit

**Affiliations:** 1 Department of Biodiversity and Conservation Biology, University of the Western Cape, Cape Town, South Africa; 2 Department of Oceanography, Dalhousie University, Halifax, NS, Canada; 3 South African Environmental Observation Network, Elwandle Coastal Node, Port Elizabeth, South Africa; Universidade de Aveiro, PORTUGAL

## Abstract

The importance of coastal upwelling systems is widely recognized. However, several aspects of the current and future behaviors of these systems remain uncertain. Fluctuations in temperature because of anthropogenic climate change are hypothesized to affect upwelling-favorable winds and coastal upwelling is expected to intensify across all Eastern Boundary Upwelling Systems. To better understand how upwelling may change in the future, it is necessary to develop a more rigorous method of quantifying this phenomenon. In this paper, we use SST data and wind data in a novel method of detecting upwelling signals and quantifying metrics of upwelling intensity, duration, and frequency at four sites within the Benguela Upwelling System. We found that indicators of upwelling are uniformly detected across five SST products for each of the four sites and that the duration of those signals is longer in SST products with higher spatial resolutions. Moreover, the high-resolution SST products are significantly more likely to display upwelling signals at 25 km away from the coast when signals were also detected at the coast. Our findings promote the viability of using SST and wind time series data to detect upwelling signals within coastal upwelling systems. We highlight the importance of high-resolution data products to improve the reliability of such estimates. This study represents an important step towards the development of an objective method for describing the behavior of coastal upwelling systems.

## 1. Introduction

Eastern Boundary Upwelling Systems (EBUS) are characterized as vast regions of coastal ocean occurring along the western shores of continents bordering the Pacific and Atlantic Oceans [1–4]. Coastal upwelling associated with EBUS is known to have a large influence on the associated ecosystem’s primary productivity, and hence the abundance, diversity, distribution, and production of marine organisms at all trophic levels [3–10]. Changes in the upwelling process over time is hypothesized to be strongly affected by anthropogenic climate change. According to the ‘Bakun hypothesis’, an increase in greenhouse gases facilitate an increase in daytime warming and night-time cooling and ultimately cause an increase in temperature gradients which will form stronger atmospheric pressure gradients [1, 11, 12]. These pressure gradients modulate the winds which ultimately affect the intensity and duration of upwelling [3, 9, 12–17]. Because changes in SST indirectly affect coastal ecosystems and have considerable, often far-reaching economic impacts [2, 3, 18–20], a better understanding of which SST products can most accurately detect upwelling will be important for any studies looking to identify and understand long-term changes to this phenomenon in EBUS [9, 15, 12, 17, 21, 22].

Previous attempts at identifying upwelling ‘events’ have employed a variety of approaches and incorporating an assortment of coastal temperature and wind variables and Ekman processes to estimate occurrences of upwelling, for example, Fielding and Davis [23] used a combination of wind speed, wind direction, and the orientation of the coast to calculate an alongshore wind component to quantify upwelling occurrences off the Western Cape coast of South Africa. Pfaff et al. [24] derived an upwelling index by contrasting offshore and onshore bottom temperatures in the southern Benguela region. Lamont et al. [25] used wind vectors to quantify upwelling variability along the same coastal region. More recently, El Aouni et al. [26] Used SST and wind data together with image processing techniques to detect and quantify upwelling signals. Several other authors made use of various other techniques to determine upwelling signals such as; Cury and Roy [27]; Demarcq and Faure [28]; Rossi et al., [29]; Benazzouz et al. [30] and Jacox et al. [31]. These examples primarily relied on wind data [11] to act as their main determinant for potential upwelling occurrences, rather than SST data. While wind patterns can act as a strong correlate for the presence of upwelling in many cases [11, 27]. SST data should arguably be more effective as these indicate presence of cold water of deep origin on the sea’s surface. However, until recently, SST data were limited in several regards concerning data quality and quantity [32–34].

SST is regarded as one of the most important variables in the coupled ocean-atmosphere system and is a particularly useful research tool in the scientific fields of meteorology and oceanography [35–42]. For over 150 years, SST data have been collected using *in situ* measurement techniques [32] with satellite measurements of SST being available since the late 1970s [43–47]. Over the past decade, techniques have been developed to allow the assimilation and blending of different SST datasets from various *in situ* and satellite platforms. These are referred to as the Level-3 and Level-4 high resolution products, with the Level-4 data being gap-free [34], and are being widely applied in studies of coastal areas [48–51]. Previous studies demonstrated that satellite-based SST data are less accurate than *in situ* data due to the complexity of the oceanic and atmospheric conditions that need to be accounted for in deriving satellite SST products [52–56] and such errors vary both regionally and temporally [57]. However, in comparison to *in situ* temperature measurements collected from ships or buoys, a major advantage of satellite SST is their global coverage and near real time availability. SST datasets with a high level of accuracy, spatial consistency and completeness, and fine-scale resolution are necessary for weather and climate forecasting and are of great importance for reliable climate change monitoring [9, 12, 17, 34, 45, 51, 58–61].

For many applications, SST data are not used or provided at the full resolution of the sensors but are averaged over defined areas to produce a gridded product [45, 62]. Gridding in this way destroys more detailed information and as a result a gridded SST measurement is taken as an estimate of the average SST across a specific grid cell over a certain time. Small-scale features can evolve during the day, but the sensor sampling during this time is not dense enough for the sub-daily global analyses at a high spatial resolution [47, 63]. Furthermore, considering that the satellites are passing overhead only once every ~24 hours, images are only captured at very specific times during the day. To capture these small-scale features in a gridded analysis, it is suggested that the development of an improved analysis would have high resolution at small-scale features in regions of good coverage and lower resolution in areas of poor coverage [47].

Here, we aimed to test the utility of a new method for detecting upwelling signals and characterizing them in terms of intensity, frequency, and duration of upwelling events in an objective manner. Our approach is analogous to the marine heatwave methodology proposed by Hobday et al. [64]—in fact, it uses the same algorithm. By assessing increases in south easterly wind with concomitant decreases in coastal SST we can more reliably estimate the likelihood of an upwelling event. Given the importance of upwelling to the coastal productivity [65, 66], regional climate, and marine ecology, the ability to measure upwelling metrics such as the frequency, duration, and intensity of upwelling signals—in addition to the occurrence of the signals itself—allows us to quantify patterns of upwelling dynamics over time, in a manner that offers the potential to link these metrics to measures of ecosystem function. Furthermore, since the resultant increase in global temperature driven by climate change has a direct influence upon increase in global SST and will also manifest in changes in the upwelling process, being able to use a variety of metrics to subject to trend analysis in upwelling will be important for ecosystem management decisions.

To this end, this study aimed to observe patterns and trends in upwelling signals in the Benguela Upwelling System (BUS) across a range of localities and spatial scales off the South African West Coast. The BUS is divided into the northern (NBUS) and southern Benguela Upwelling Systems (SBUS) by a zone of intense perennial upwelling activity in Lüderitz within the Namibian region [25, 26, 67–69]. Meteorologically these regions are distinct. In the south, wind- induced upwelling reaches a maximum during spring and summer, whereas the northern region exhibits relatively less seasonal variation [67, 70–72]. Coastal upwelling commonly occurs between Cape Agulhas, in the south, to southern Angola in the north. We selected the SBUS upwelling system for this study because this physical process provides a strong seasonal signal of increasing and decreasing SST that is strongly localized to known centers of upwelling, and which relates to the coastal wind field that drives the offshore advection of water mass [71–73]. We apply our new method for identifying upwelling signals to data representative of this region. Because upwelling is such a well-characterized oceanographic process, the resultant fluctuating SST signal should be observed across independent SST products. Here we assess blended SST products covering a range of spatial grid resolutions from 0.05° × 0.05° to 0.25° × 0.25°. We hypothesized that the higher resolution data should have a greater fidelity at detecting these upwelling signals, some of which might only be confined to smaller spatial scales or localized closer to the shore.

## 2. Materials and methods

### 2.1. Site description

**Fig 1:**
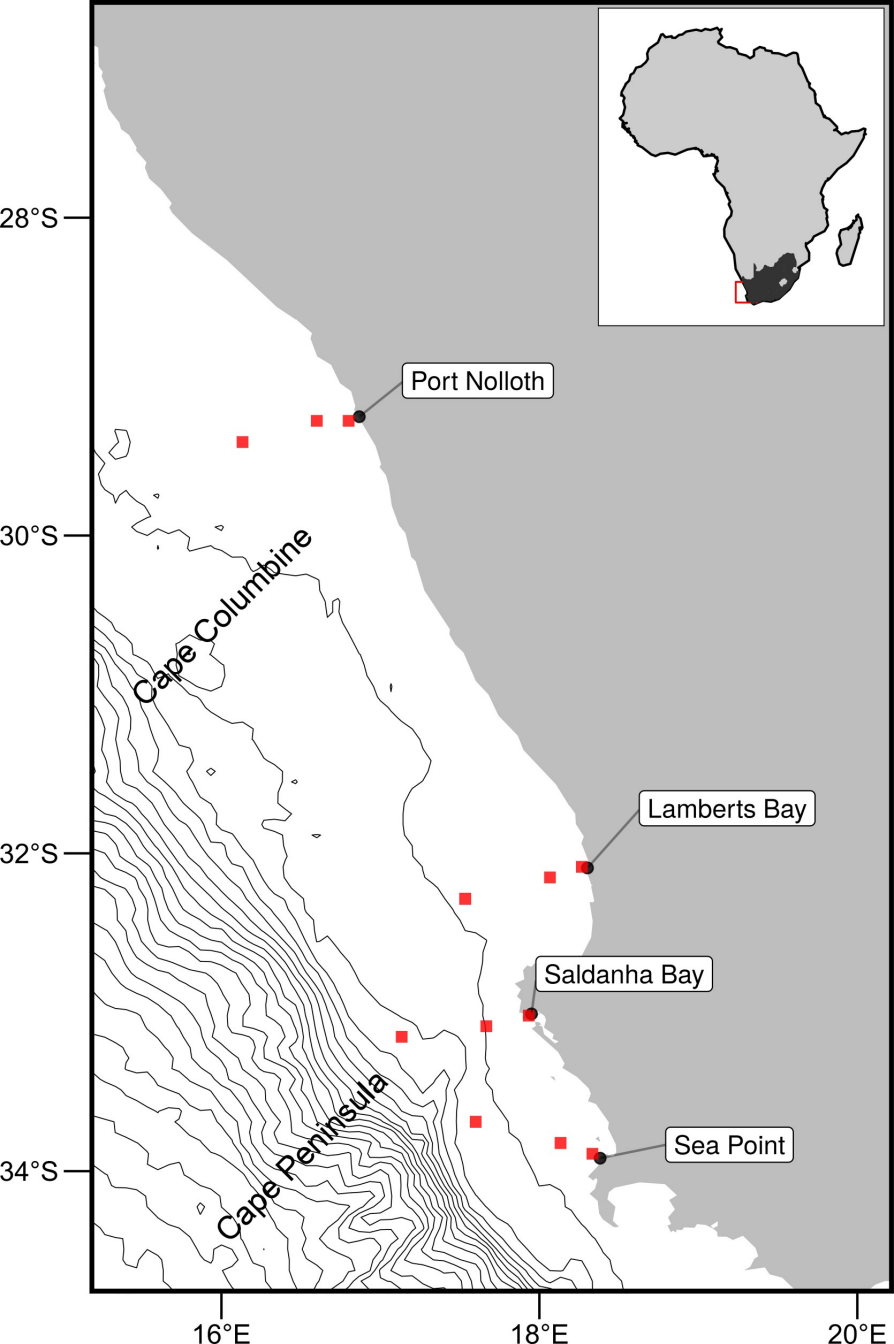
Map of the western portion of southern Africa showing the coastal bathymetry of the southern BUS. The black points represent the location of the *in situ* temperature recorders, and the red boxes show the pixels used along the shore normal transect from the satellite sea surface temperatures (SST) time series. The red boxes are at 0 km, 25 km and 50 km from the shoreline.

The western region of the South African coastline is dominated by the Benguela Current, which forms the foundation of the Benguela Upwelling System (BUS) [74], and provides a natural laboratory for this study. Seasonal upwelling is controlled by south-easterly trade winds, with intense upwelling occurring throughout the summer months. This creates distinct temperature variations with much lower temperatures within the upwelling cells over a narrow continental shelf from the Cape Peninsula to Cape Columbine. To assess upwelling within the BUS, four sites from the South African Coastal Temperature Network (SACTN) dataset [61, 75] were selected as points of comparison (see below). Each site was situated along the West Coast of South Africa, and shore normal transects were used to sample the data at 0, 25 and 50 kms (Fig 1). Where 0 km pixels were those closest to their corresponding *in situ* site.

Upwelling processes in the southern Benguela are highly influenced by bottom topography [76]. The continental shelf that forms the eastern boundary of the Cape Basin, defined roughly by the 200 m isobath, varies in width from 10 km at prominent capes to 150 km near Port Nolloth. In the vicinity of the Cape Peninsula and Cape Columbine, the coastline is irregular, and two canyons associated with these features cut into the shelf, parallel to the coast [76]. The dynamic topography of the area is such that the Agulhas Current water is fed into the Benguela systems from south of the Agulhas Bank. Upwelling in the BUS occurs in several distinct upwelling cells that form at locations of maximum wind stress curl, and where there is a change in the orientation of the coastline. Lutjeharms and Meeuwis [77] distinguished eight different cells: Cunene, Namibia, Walvis Bay, Lüderitz, Namaqua, Columbine, Cape Peninsula, and the Agulhas cell. Shannon and Nelson [78] included three more upwelling cells along the south coast. Given that this research study is restricted to the southern Benguela, discrete upwelling cells at Cape Columbine and the Cape Peninsula will be discussed [76]. The Cape Columbine and Cape Peninsula upwelling cells are identified as two distinct bands of cold water on the inner and mid-continental shelves at a depth of 0–100 m, where upwelling is generally more intense during summer [76]. This cold water is apparent along the length of the inner (0–100 m) and mid-continental (100–200 m) shelves [79]. In the Cape Peninsula region, a change in Sea Surface Temperature (SST) is present at Port Nolloth notably owing to the combined effects of being at the point of the southern limit of the Cape Peninsula upwelling cell and the sudden broadening of the inner shelf immediately to the south of the Peninsula.

### 2.2. Datasets

This study uses four Level-4 remotely sensed temperature datasets compiled by several organizations. Product 1 is the AVHRR-only (Advanced Very High-Resolution Radiometer) Optimally Interpolated Sea Surface Temperature (OISST) dataset, which has been providing global SST for nearly four decades [80]. OISST is a global 0.25° × 0.25° gridded daily SST product that assimilates both remotely sensed and in situ sources of data to create a gap-free product [81]. The second product is the Group for High Resolution Sea Surface Temperature (GHRSST) Canadian Meteorological Center (CMC) Level-4 0.2° × 0.2° version 2; it combines infrared satellite SST at numerous points in the time series from the AVHRR, the European Meteorological Operational-A (METOP-A) and Operational-B (METOP-B) platforms, as well as the microwave SST data from the Advanced Microwave Scanning Radiometer 2 in conjunction with in situ observations of SST from ships and buoys from the ICOADS program. The third dataset is the Multi-scale Ultra-high Resolution (MUR) SST Analysis, which is produced using satellite instruments with datasets spanning 1 June 2002 to present times. MUR provides SST data at a spatial resolution of 0.01° × 0.01° and is currently among the highest resolution SST datasets available. The final dataset is the GHRSST analysis produced daily using a multi-scale two-dimensional variational (MS-2DVAR) blending algorithm on a global 0.01° grid known as G1SST. This product uses satellite data from a variety of sensors, such as AVHRR, the Advanced Along Track Scanning Radiometer (AATSR), the Spinning Enhanced Visible and Infrared Imager (SEVIRI), the Moderate Resolution Imaging Spectroradiometer (MODIS), and in situ data from drifting and moored buoys. We acknowledge that not all products are completely independent as they share the use of AVHRR SST data, but the amount of subsequent blending, the incorporation of other SST data sources, the different blending and interpolation approaches used, and the differing final grid resolutions make them acceptably different for this study.

These SST products are compared against in situ temperature records from the South African Coastal Temperature Network (SACTN). This dataset consists of coastal seawater temperatures at 129 sites along the South African coastline, measured daily from 1972 until 2017 [61, 75]. Of these, 80 were measured using hand-held thermometers and the remaining 49 were measured using underwater temperature recorders (UTRs). For this analysis, the data were combined and formatted into standardized comma separated values (CSV) files which allowed for a fixed methodology to be used across the entire dataset. In situ SST measurements were collected using a thermometer at a depth of 0 m for the four sites used in this study. The objective of this study was to identify upwelling signals using a variety of separate SST products for the period between 2011-01-01 to 2016-12-31. We specifically selected this range of years as they provide a sufficient overlap in time series between four remotely sensed SST and in situ datasets thereby offering candidate years for points of comparison.

An advantage to using in situ data over satellite data is that they may provide a more realistic representation of the thermal properties closer to the coast, whereas satellite data fail to accurately capture and represent temperature properties within the same spatial context. The result is that in situ data may be better at explaining upwelling signals within the coastal inshore environment. Further, evidence by Smit et al. [54] has shown that satellite data along the South African coastline may have a warm bias as much as 6°C greater than in situ temperatures within the nearshore. Time series for each of the remotely sensed SST data products were created at the nearest pixel to each in situ station, and at each pixel along the shore-normal transects from these stations at 25 and 50 km from the coast (Fig 1). Wind speed and direction data were provided by the South African Weather Service (SAWS) at a three-hour resolution. The wind stations closest to each of the in situ stations were used to calculate the upwelling index (see below).

### 2.3. Defining and detecting upwelling

To detect and analyze upwelling at the four sites within the BUS, it was first necessary to define when upwelling occurred. To accomplish this, a set of threshold values for identifying when the phenomenon was taking place was required. For the wind component, we parsed alongshore, wind events at each site. We limited this to only include alongshore winds stronger than 5 m.s^*-1*^ [11, 27]. since upwelling tends to only occur when wind exceeds the above speeds. We then used several parameters of those winds to inform an upwelling index calculated using the formula presented by Fielding and Davis [32]:

upwellingindex=μ(cosθ−160)

where μ represents the wind speed (m/s), θ represents the wind direction in degrees, and 160 is the orientation of the west coast in degrees [82]. The above equation produces a value called the ‘upwelling index’. An upwelling index < 0 represents downwelling whilst an upwelling index > 0 represents upwelling [32]. For the temperature component, we evaluated coincidental drops in SST at each site when the upwelling index was greater than 0. If temperature dropped to the seasonally varying 25^*th*^ percentile of SST for a particular site, we deemed this as confirmation of the occurrence of an upwelling event at that site. See Schlegel et al. [61] for a similar threshold used to detected marine heatwaves and coldspells. with these thresholds established, it was then necessary to identify the number of consecutive days that must be exceeded for an upwelling signal to qualify as a discrete event. It must be noted that upwelling is known to vary on a seasonal basis and may also occur hourly (sub-daily). Therefore, the minimum duration for the classification of an upwelling signal was set as one day, the rationale being that data from the SACTN dataset as well as the satellite remotely sensed SST data are collected only at a daily resolution, preventing a temporally finer definition. With the upwelling index, SST data, and duration for an upwelling signal established, the detect_event() function from the **heatwaveR** package [83] was used to calculate metrics for the upwelling signals. Because upwelling signals were calculated relative to percentile exceedances, rather than a fixed temperature threshold, upwelling signals could occur any time of the year; however, upwelling was shown to be more dominant during summer months (December, January, and February), as expected. This method of determining upwelling signals is novel as it considers both SST and wind parameters, and provides us with a descriptive statistical output, which include three metrics that define the properties of each of the signals detected (Table 1).

**Table 1:** Metrics of upwelling signals and their descriptions.

Name (unit)	Definition
Count (*n*)	Number of upwelling signals per year
Mean intensity (°C)	Mean temperature anomaly during the upwelling signal
Cumulative intensity (°C.days)	Sum of the daily intensity anomalies over the duration of the signal

ANOVAs were used to compare the upwelling metrics against three main effects: *site*, *product*, and *distance*. Upwelling metrics as a function of satellite product type were assessed using *product* as the main effect, and nesting *distance* within *site*. To establish whether differences existed between sites or distances from the shore, the upwelling metrics were assessed as a function of *site* or *distance* independently for each satellite. Restrictions to experimental design prevented testing interaction effects within *product* types. These analyses sought to test if significant differences occurred between sites and data products. A Pearson product moment correlation was used to identify if the same upwelling signal detected at 0 km from the coastline were also regularly detected at 25 and 50 km from the coastline. The signals were classified by start and end date within the same data product. Thereafter, the average numbers of upwelling signals detected by each individual data product across all sites were compared using an ANOVA test. Thereafter, a Chi-square analysis was used to compare of the number of upwelling signals detected when including and excluding an SST filter when determining upwelling signals.

## 3. Results

**Fig 2:**
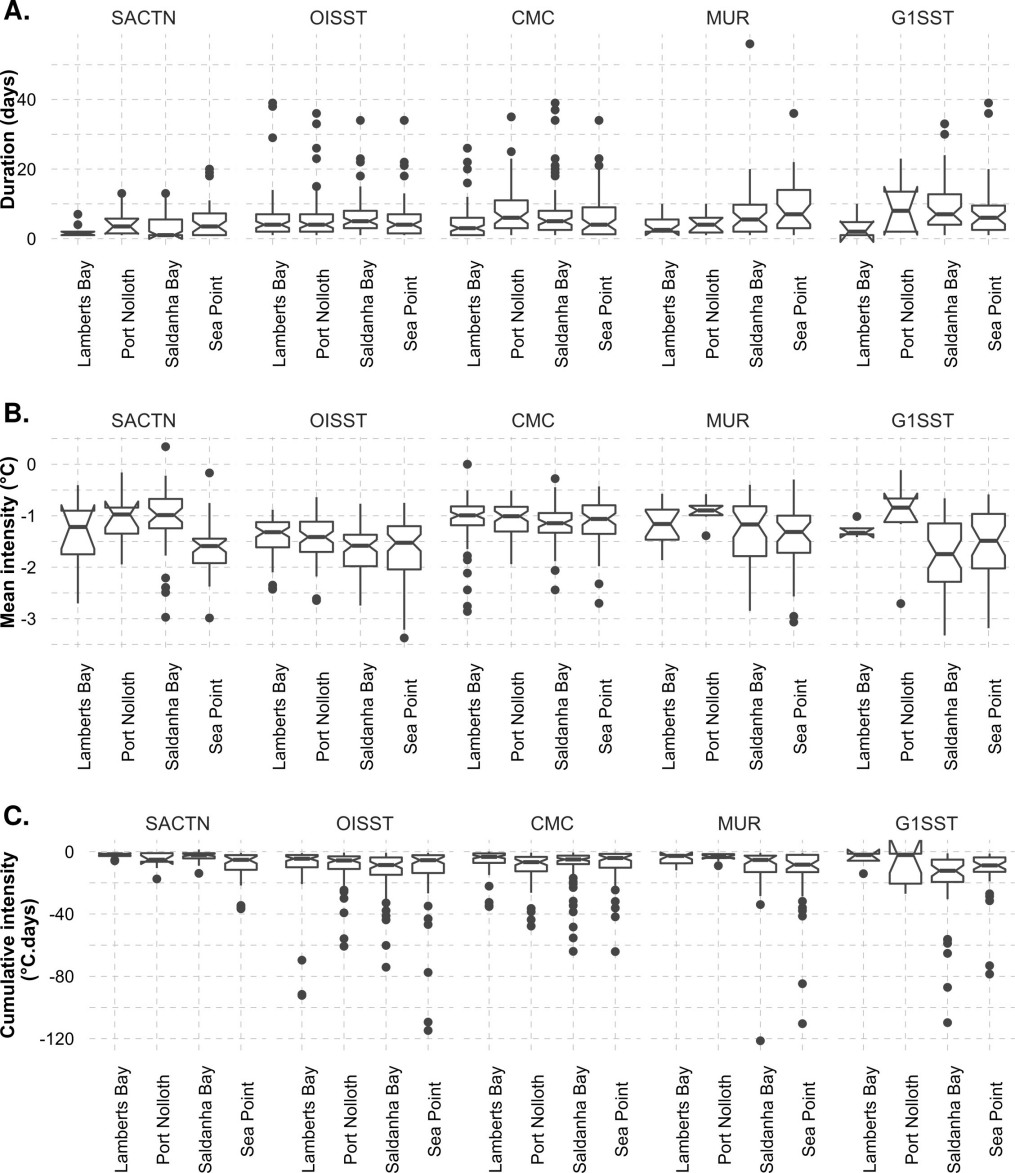
Boxplots showing the upwelling A) duration, B) mean intensity, and C) cumulative intensity for the upwelling signals detected with the four satellite products and the SACTN *in situ* collected data at the different sites during summer months (December, January, and February), over a six-year period. The lower and upper hinges correspond to the first and third quartiles, and outliers are shown as points. The notches offer a guide to significant difference in medians, *i*.*e*., if the notches of two box plots overlap it suggests that there is no statistically significant difference between the medians being compared.

One-way ANOVA indicated no significant difference in upwelling duration between sites across each respective data product: SACTN (d.f. = 3, *F* = 5.91, *p* > 0.05), OISST (d.f. = 3, *F* = 0.12, *p* > 0.05), CMC (d.f. = 3, *F* = 0.57, *p* > 0.05), MUR (d.f. = 3, *F* = 2.50, *p* > 0.05) and G1SST (d.f. = 3, *F* = 0.64, *p >* 0.05) (Fig 2A) products. The Sea Point site displayed the longest mean duration of upwelling signals. Lamberts Bay had the shortest duration upwelling signals. Particularly, the Lamberts Bay data from the SACTN dataset showed the shortest duration upwelling signals.

A significant difference was found in mean intensity of upwelling between sites in the OISST (d.f. = 3, *F* = 5.82, *p* < 0.001) and SACTN (d.f. = 3, *F* = 7.39, *p* < 0.001) products. Conversely, no significant difference was found in the CMC (d.f. = 3, *F* = 1.04, *p* > 0.05), MUR (d.f. = 3, *F* = 2.48, *p* > 0.05) and G1SST (d.f. = 3, *F* = 2.66, *p* > 0.05) products (Fig 2B). There was no significant difference in cumulative intensity of upwelling between sites in the CMC (d.f. = 3, *F* = 0.58, *p* = 0.62) (Fig 2C). The mean intensity of upwelling signals was highest in Saldanha Bay and Sea Point for the MUR and G1SST data. We found that there was a significant difference between cumulative intensity of upwelling signals between sites only when using the SACTN dataset. The cumulative intensity of upwelling signals was most intense in Saldanha Bay and Sea Point for all of the products.

**Fig 3:**
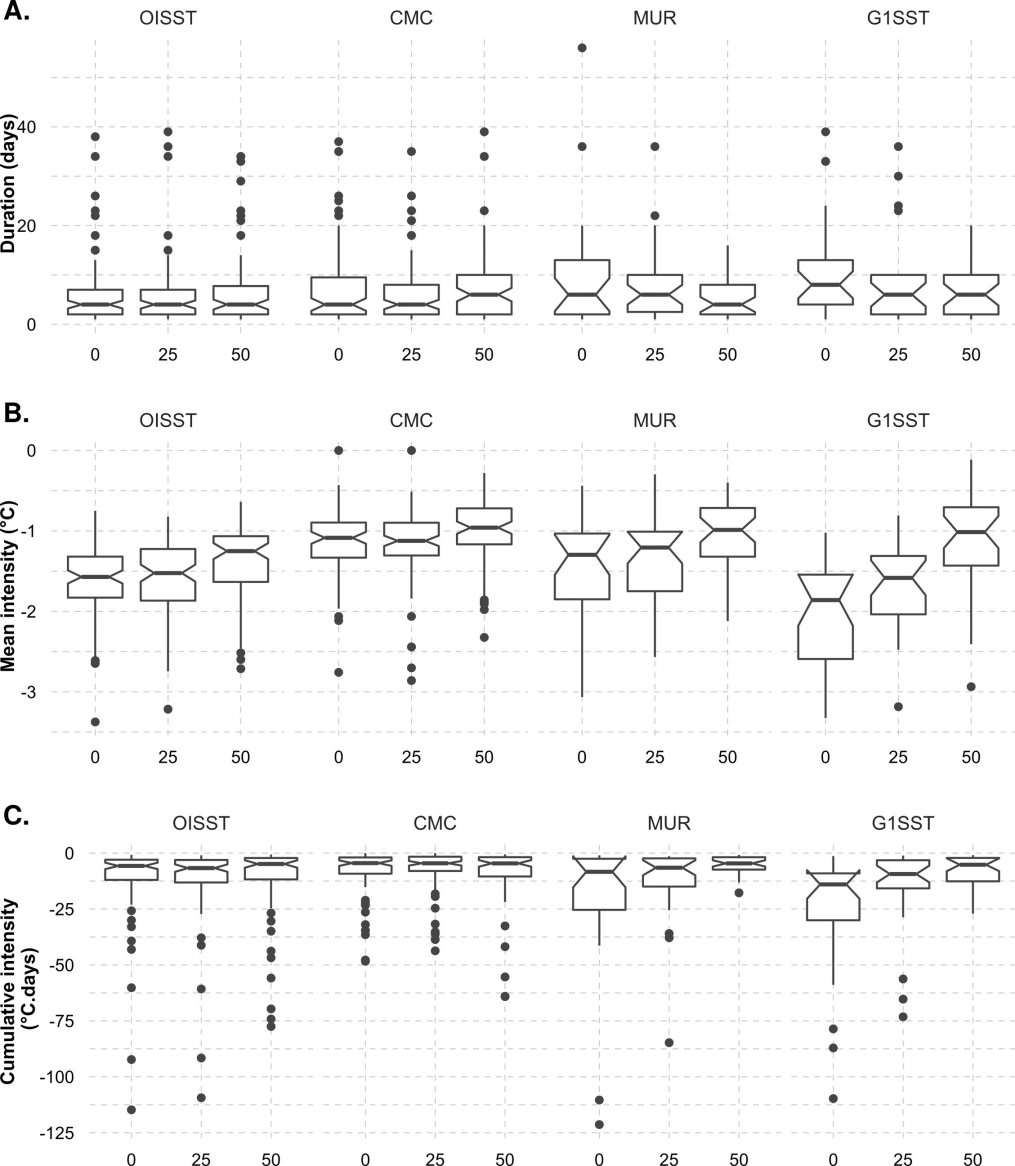
Boxplots showing the A) duration, B) mean intensity, and C) cumulative intensity for each of the upwelling signals detected at various distances (km) from the shore for the four satellite products during summer months (December, January, and February), over a six-year period. The properties of the boxplots are as in Fig 2.

An ANOVA showed no significant difference in the duration of upwelling signals detected at different distances from the shore during the summer season in the CMC (d.f. = 2, *F* = 1.03, *p* = 0.35) and G1SST (d.f. = 2, *F* = 2.55, *p* > 0.05) products. However, a significant difference was present across the MUR (d.f. = 2, *F* = 3.33, *p* < 0.05) and OISST data (d.f. = 2, *F* = 5.17, *p* < 0.05) products. The MUR and G1SST often yielded the longest duration of upwelling signals at 0 and 25 km from the shore (Fig 3A).

Significant differences in the mean intensity of upwelling signals were present across different distances from the shore in the G1SST (d.f. = 2, *F* = 15.38, *p* < 0.001), MUR (d.f = 2, *F* = 5.12, *p* < 0.001) and OISST (d.f. = 2, *F* = 5.17, *p* < 0.05). MUR and G1SST products displayed the highest mean intensity of upwelling signals at 0 km from the coast (Fig 3B). The mean intensity of upwelling decreased further away from the coast in the higher resolution products.

A one-way ANOVA showed a significant difference in the cumulative intensity of upwelling signals detected at different distances from the shore in the G1SST (d.f. = 2, *F* = 7.03, *p* < 0.05) and MUR (d.f. = 2, *F* = 4.69, *p* < 0.05) data products. (Fig 3C). The CMC (d.f. = 2, *F* = 0.33, *p* > 0.05) and OISST (d.f. = 2, *F* = 0.06, *p* > 0.05) products showed no significant difference in cumulative intensity. The OISST, MUR and G1SST products yielded the highest cumulative intensity at 0 km from the coastline. The cumulative intensity of upwelling signals for all products decreased further from the coast. The results of a nested ANOVA showed that there was a significant difference in the duration of upwelling signals detected amongst the data products (nested ANOVA, d.f. *=* 3, *F* = 3.01, *p* < 0.02). The G1SST product had the longest duration of upwelling signals while the OISST products had the shortest. We found a significant difference in the mean intensity of upwelling signals between data products (nested ANOVA, d.f. = 3, *F* = 49.93, *p* < 0.001). The G1SST and MUR data products showed the highest mean intensity while CMC had the lowest. We also found a significant difference in the cumulative intensity of upwelling signals between the data products of different resolutions (nested ANOVA, d.f. = 3, *F* = 5.71, *p* < 0.05). The G1SST product showed the strongest cumulative intensity of upwelling and the CMC data the weakest.

Pearson correlation revealed the possibility of observing the same upwelling signal detected at 0, 25, and 50 km from the coast respectively varied across the individual data products at each of the four sites (Table 2). Overall, we found that upwelling occurred simultaneously at 0 km and at 25 km considerably more frequently than between 0 km and 50 km from the coastline. In addition, the likelihood of detecting upwelling signals at 50 km from the coastline were notably lower throughout all pairwise comparisons. The individual data products yielded different counts of upwelling signals at distances of 0 km, 25 km, and 50 km from the coastline. There was no significant difference between the number of upwelling signals collected at the different sites (one-way ANOVA: F = 1.73, d.f = 3, SS = 520, p > 0.05). However, there was a significant difference in the number of signals detected between products (F = 146.611, d.f = 3, SS = 40638, p < 0.001) and at different distances from the coastline (F = 0.76, d.f = 2, SS = 141, p > 0.05).

**Table 2:** A Pearson correlation of the relationship between the number of signals detected at 0 km versus a distance of 25 km and between the number of signals at 0 km and 50 km.

Product	Site	0 km vs 25 km	0 km vs 50 km
**OISST**	Port Nolloth	0.97 ****	0.52 ***
	Lamberts Bay	0.36	0.21*
	Saldanha Bay	0.91 ****	0.51***
	Sea Point	0.95 ***	0.73 **
**CMC**	Port Nolloth	0.95***	0.60**
	Lamberts Bay	0.48*	0.30**
	Saldanha Bay	0.47 ***	0.38**
	Sea Point	0.75 ****	0.33 ***
**G1SST**	Port Nolloth	0.75 ***	0.76***
	Lamberts Bay	0.68 **	0.57 **
	Saldanha Bay	0.44 **	0.33 *
	Sea Point	0.47 **	0.32 **
**MUR**	Port Nolloth	0.92 **	0.80
	Lamberts Bay	0.86 ****	0.76 **
	Saldanha Bay	0.70 *	0.62 *
	Sea Point	0.87 **	0.72 *

Significant levels are as follows

* p ≤ 0.05

** p ≤ 0.01

*** p ≤ 0.001, and

**** p ≤ 0.0001.

Comparisons of the number of upwelling signals detected when including and excluding SST data revealed that significantly more upwelling events were present across sites and data products when using only wind data (Table 3; *χ*^2^ = 141.18, *p* < 0.001). The results of Chi-squared test comparing the mean number of upwelling events between filtered and non-filtered counts per data product showed that on average the filtered data had lower numbers of upwelling events than expected when assessing each dataset individually. However, these differences in the count of upwelling events were only significant in all of the products (Table 3). Similarly, site-specific comparisons revealed that upwelling events at all sites showed significant differences between filtered and unfiltered counts of upwelling events, with unfiltered counts being notably higher in all cases.

**Table 3:** Results of Chi-squared test comparing the numbers of upwelling signals detecting with and without SST as a filter across the four data products at four sites.

Comparison	*χ*2	*p*	d.f.
**All products and sites**	141.18	<0.001	1
**OISST**	15.77	<0.001	3
**MUR**	14.39	0.002	3
**G1SST**	20.04	<0.001	3
**CMC**	20.47	<0.001	3
**SACTN**	34.11	<0.001	3
**Lamberts Bay**	108.77	<0.001	4
**Port Nolloth**	152.45	<0.001	4
**Saldanha Bay**	90.41	<0.001	4
**Sea Point**	19.65	<0.001	4

## 4. Discussion

### 4.1. Detection of upwelling signals

Over the past few decades, upwelling has been mainly described and determined in general terms using a variety of upwelling indices derived from diverse combinations of wind, SST, and Ekman transport variables [2–26, 29–31, 84]. We demonstrate that our novel approach to characterize upwelling events using SST in combination with wind variables to determine metrics that objectively and quantitatively describe the upwelling process offers a similarly versatile means for detecting changes in upwelling dynamics associated with climate change. We calculate a set of summary statistics (i.e., the metrics) for each upwelling ‘signal,’ including its intensity, duration, and frequency by making use of the marine heatwave algorithm [61, 64]. Time series of these metrics are intuitively understood and allow for upwelling signals to be uniquely described and compared across space and time, even between upwelling regions. The use of this approach is not independent on the nature of the data, and here we explore this for SST.

### 4.2. Data products

Our analysis showed that differences exist between SST products and sites when comparing the upwelling metrics. The highest resolution data, MUR and G1SST, which are available on a 0.01 grid, yielded the longest duration and cumulative intensity of upwelling signals compared to the coarser resolution data products. The MUR product consistently yielded upwelling signals of the greatest intensity. Upwelling signals were most intense at the shore in all the SST products. Analysis of the CMC and SACTN datasets revealed that signals did not often exceed a duration of 10 days, whereas in OISST, MUR and G1SST the signals were detected for up to 14 days and even longer in some rare cases. Moreover, most of the signals detected in CMC and SACTN products only lasted for three days. This was similar for the higher resolution data products (G1SST and MUR) which also showed a high prevalence of signals lasting for just four days. In most cases, the number of signals detected at 0 km was higher than the number of signals detected at 50 km for the data products with the highest resolution. We also noted differences in mean intensity between products and distances from the site. The highest number of signals detected were recorded in the OISST and CMC products. The results show that the use of wind data without corresponding SSTs is likely to produce exaggerated estimations of upwelling. However, by incorporating SST data allows for a greater chance of reducing type I errors, i.e., false positives for estimating upwelling and reducing the overall likelihood for erroneously claiming an upwelling event based on wind data alone when corresponding SST are not cooling.

Level-4 gridded SST datasets obtained from satellite imagery have provided an important understanding of offshore oceanographic processes. Their utility often stems from the fact that they are spatially complete. However, coastal features such as upwelling cells are often smaller than the highest resolution of most SST products [54]. In this study, estimates of upwelling duration, mean intensity and cumulative intensity may have been overestimated from data collected by the MUR and G1SST data products when comparing them to the in situ collected SACTN data. These products are more likely to be susceptible to errors relating to limitations and data collection biases associated with satellite-derived sampling [85, 86]. The overestimated metrics of upwelling may be due to errors from different sources which are produced at each of the successive data processing level [86]. SST accuracy refers to the retrieval error produced at Level-2 (derived SSTs at pixel bases), but Level-3 (binned, gridded, and averaged Level-2 values) and Level-4 fields are extensively used in climate and modeling studies, mainly because of the desirable features of being “gridded and gap-free” [86].

It is important to note that the data sources are intrinsically different in the ways in which they were obtained or recorded. Consequently, discrepancies between datasets are to be expected. For example, the SACTN in situ collected data will reflect the actual temperature of the water being measured but instrumental differences when using a thermometer or an electronic sensor will result in inconsistencies. This is particularly prevalent because satellite temperatures are collected remotely, and sensors do not contact the water. Smit et al. [54] showed that warm and cold biases exist along the southern and western coastal region of South Africa, and the juncture between upwelling and non-upwelling regions tend to influence the variability and magnitude of the SST bias. While flagging techniques are supposed to occasionally flag ‘good’ values [87], it was found that flagging may occasionally be too vigorous for EBUS [88]. For example, the flagging method used on an OISST reference test induces warm coastal bias in data from both the MUR and G1SST data during summer [88]. It should be noted that this phenomenon can be explained by strong coastal SST gradients in these upwelling regions—here pixel-based corrections developed for oceanic applications often fail or are inappropriate due to the strong thermal gradients associated with upwelling.

Flagging techniques used to de-cloud data are also known to reduce strong biases at a monthly scale with strong horizontal SST gradients especially in upwelling systems [54]. Missing pixels at the land/sea edge or ‘land bleed’—i.e., pixels not flagged as missing, but which are influenced by land temperatures ‘mixing’ with the actual sea temperatures, may also influence temperature data obtained. Contributing towards the magnitude of differences in upwelling signals detected between the different SST products are factors such as data resolution, proximity from the coastline, and the presence or absence of upwelling cells or embayments.

SST generally shows a high degree of correspondence with measurements obtained by buoys and other sources of in situ seawater temperature measurements [54, 89]. However, although SST products developed offshore and within the open ocean are being applied to the coastal regions, reports exist to inform users to exercise caution when using SST datasets in these coastal regions [90]. Many upwelling pulses may be localized and of short duration (i.e., lasting for a few hours or days; Duncan et al. [91], Sawall et al. [92]), which may contribute to the higher resolution (MUR and G1SST) products yielding more signals lasting for a longer period when compared to the coarser resolution products (e.g., OISST). Prior investigations for quantifying the durations of upwelling events across the globe have adopted several approaches and estimates derived using various methodologies. For example, Wang et al. [93] used wind driven Ekman transport indices to estimate that upwelling events in the southern hemisphere last fewer than 10 days on average. Contrastingly, Iles et al. [94] used PFEL indices to estimate upwelling duration as > 6 days. Here we estimate upwelling as only lasting for 3–6 days on average, considerably shorter than previous estimates elsewhere. Both MUR and G1SST have a limited time series length (MUR: 2002-Jun-01 to Present, G1SST: 2010-Jun-09 to 2019-Dec-09) and for this reason are not well suited to climate change studies, which require time series of at least 30 years in duration. In this case, the OISST dataset would be more suitable. The adoption of a consistent definition and metrics for upwelling will facilitate comparisons between different upwelling signals, across seasons and at regional scales. It will also facilitate the comparison of observed signals against modelled projections, which will be useful in understanding future changes in upwelling signals. Confidence in the robust detection of upwelling signals will only be achieved with the use of high-quality datasets and a verifiable method.

### 4.3. Oceanography

At the latitude of the Cape Peninsula, cooler upwelled water (<14°C) is confined primarily to the narrow inner shelf and this is evident in our data as we observe the most intense upwelling signals closer to the shore. It is also evident that the high resolution G1SST and MUR data sampled in Lamberts Bay, Saldanha Bay and Sea Point show the highest number of upwelling signals detected at the narrow inner shelf with fewer signals collected at the mid latitude shelf. Our findings further show that the coarser resolution (OISST) product fails to detect signals further offshore, as seen in Sea Point. Currie [95] and Hart and Currie [96] further explain that the BUS consists of a series of anticyclonic eddies of interlocking cool and warm water, which is in a constant state of change. This allows for upwelling cells or patches, formed by water that originates from between 200 and 300 m deep, to not be uniform along the coast. By understanding the topography, it is evident that, although upwelling is not visible at the surface, subsurface upwelling is possible [76]. This further suggests that in cases when the same signal was detected at the shoreline and 25 km from the coast, a corresponding signal would not be identified at 50 km and this may be explained by sub-surface upwelling.

While the SST data may be satisfactory for interpretation of regional phenomena, they nevertheless suffer from several drawbacks when applied within the coastal region. Here the interaction of hydrodynamic and atmospheric forces creates a complex system which is influenced by larger variability at smaller spatial scales than further offshore [88]. Hydrodynamic regimes, such as stratified water columns, may break down at the coast in very shallow waters, and seawater temperatures measured there may not directly relate to SSTs sampled further from the coast at the ocean’s surface [97]. This inshore hydrodynamics may be described by a) the injection of turbulence through breaking waves, thus increasing the breakdown of the mixed layer; b) convective mixing due to the cooling through the process of evaporation, which occurs during winter months under cool dry air; c) tidal mixing which minimizes the vertical thermal gradient; and d) mixing through velocity often caused by wind driven currents. Together, these processes homogenize the first few meters of the water column and therefore minimize the difference between the surface temperature and deeper bulk temperature [98]. In hydrodynamically active zones, such as the BUS, the absence of shallow stratification would cause a portion of cooler water than the bulk surface waters of the ocean to which satellite SSTs have been referenced. Thermal heating of coastal waters may also be exaggerated due to the proximity to the coast [88]. This type of heating is commonly seen in embayments, which reduce water exchange and limit wave activity and ultimately affect the deepening of the thermocline. These processes are highly variable on a spatial and temporal scale depending on the coastal bathymetry and wind regime.

## 5. Conclusions

Overall, in the rapidly changing climate, the detection, characterization, and prediction of upwelling signals will become increasingly important. The impact of climate change on upwelling is an emerging area of interdisciplinary research with potential for collaborative initiatives in understanding coupled phenomena across physical oceanographic, ecological, and socio-economic areas of inquiry. The metrics of upwelling that we introduce here—intensity, duration, and frequency of signals of upwelling—provide a consistent framework that lends itself to be quantitatively coupled to metrics of change indicative of aspects of the regional biology, ecological impacts, and trends in the societal aspects of stakeholders whose livelihoods and businesses are coupled with the functioning of upwelling systems. Our approach not only provides us with a new method of detecting upwelling signals, which is useful to observe trends in upwelling signals over time, but also emphasizes the importance of selecting the correct data product in concert with knowledge about the nature of the physical phenomena being studied.
